# Association of sleep disordered breathing with clinical trajectories in patients undergoing cardiac surgery

**DOI:** 10.1186/cc12433

**Published:** 2013-03-19

**Authors:** J Roggenbach, B Tan, E Von der Leyen, A Weymann, M Karck, E Martin, S Hofer

**Affiliations:** 1 University of Heidelberg, Germany

## Introduction

The prevalence of sleep disordered breathing (SDB) is presumably high among individuals with cardiac diseases [1], nonetheless SDB remains predominantly undiagnosed. However, unrecognized SDB might have relevant impact on the postoperative course of patients undergoing cardiac surgery [2].

## Methods

Polygraphic recordings of 181 patients, without previous diagnosis of SDB, undergoing standard cardiac surgical procedures with extracorporeal circulation were obtained during a preoperative night. The apnea-hypopnea index (AHI - the number of apneas, hypopneas per hour recorded) was determined and compared with clinical characteristics and postoperative course.

## Results

The prevalence of SDB was considerably high among all examined patients. Median AHI was 20.8 (interquartile range, 10.6 to 36.4). Preoperative AHI was >30 in 32% of all examined individuals. During the first three postoperative days, preoperative AHI >30 was associated with a prolonged weaning time, a reduced oxygenation index (arterial pO_2_/FiO_2_), an impaired kidney function, an augmented inflammatory response and an overall increased length of stay in the ICU. The observed association of high preoperative AHI values with postoperative clinical characteristics remained statistically significant throughout the first three postoperative days.

## Conclusion

Undiagnosed SDB is highly prevalent among cardiac surgical patients. Clinical trajectories of individuals with severe SDB are described by a prolonged recovery of pulmonary function, delayed weaning and a pronounced inflammatory response after surgery. Screening for SDB might identify patients that are susceptible for a complicated postoperative course.
